# Shifts in plant functional community composition under hydrological stress strongly decelerate litter decomposition

**DOI:** 10.1002/ece3.6310

**Published:** 2020-04-29

**Authors:** Julia Walter, Carsten M. Buchmann, Frank M. Schurr

**Affiliations:** ^1^ Institute of Landscape and Plant Ecology University of Hohenheim Stuttgart Germany

**Keywords:** biomass‐ratio hypothesis, community‐weighted mean functional traits, drought, effect traits, functional diversity, litterbag, niche‐complementarity hypothesis, pulsed stress, response

## Abstract

Litter decomposition is a key process of nutrient and carbon cycling in terrestrial ecosystems. The decomposition process will likely be altered under ongoing climate change, both through direct effects on decomposer activity and through indirect effects caused by changes in litter quality. We studied how hydrological change indirectly affects decomposition via plant functional community restructuring caused by changes in plant species’ relative abundances (community‐weighted mean (CWM) traits and functional diversity). We further assessed how those indirect litter quality effects compare to direct effects. We set up a mesocosm experiment, in which sown grassland communities and natural turf pieces were subjected to different hydrological conditions (dryness and waterlogging) for two growing seasons. Species‐level mean traits were obtained from trait databases and combined with species’ relative abundances to assess functional community restructuring. We studied decomposition of mixed litter from these communities in a common “litterbed.” These indirect effects were compared to effects of different hydrological conditions on soil respiration and on decomposition of standard litter (direct effects). Dryness reduced biomass production in sown communities and natural turf pieces, while waterlogging only reduced biomass in sown communities. Hydrological stress caused profound shifts in species’ abundances and consequently in plant functional community composition. Hydrologically stressed communities had higher CMW leaf dry matter content, lower CMW leaf nitrogen content, and lower functional diversity. Lower CWM leaf N content and functional diversity were strongly related to slower decomposition. These indirect effects paralleled direct effects, but were larger and longer‐lasting. Species mean traits from trait databases had therefore considerable predictive power for decomposition. Our results show that stressful soil moisture conditions, that are likely to occur more frequently in the future, quickly shift species’ abundances. The resulting functional community restructuring will decelerate decomposition under hydrological stress.

## Graphical abstract

We show for the first time not only that community‐weighted mean functional traits and functional diversity rapidly change under hydrological stress (dryness and waterlogging), but also that these shifts strongly decelerate litter decomposition. These indirect effects on litter decomposition via changes in litter quality parallel direct effects on decomposer activity, but are even larger in magnitude and last longer. To our knowledge, this is the first study showing a link between effects of hydrological conditions on functional community structure with consequences for an ecosystem process, such as litter decomposition.

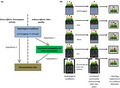

## 1 INTRODUCTION

Climate change alters precipitation and temperature regimes worldwide, thereby causing hydrological change in many plant communities (IPCC, 2018). Extended dry periods might become more frequent in the future, but also average precipitation is likely to increase over many areas (IPCC, 2018; Orth, Zscheischler, & Seneviratne, 2016). Extreme rainfall events have already been observed to increase and are projected to increase further during the 21st century for many regions, contributing to an increase in flooding risk (IPCC, 2012, 2018).

Plant litter decomposition is a key ecosystem process, recycling nutrients and carbon (C) from dead organic matter. It consequently regulates soil nutrient availability as well as soil C sequestration and turnover, feeding back on primary productivity and atmospheric chemistry. Grasslands store 10% to 30% of the world's total soil C (de Deyn, Cornelissen, & Bardgett, 2008; Risch, Jurgensen, & Frank, 2007), and temperate grasslands globally sequester about 0.21 Gt C per year (Grace, 2004). Understanding how litter decomposition in grassland is modified by ongoing climate change is therefore of major importance, because changes to the decomposition process can change soil C emissions and therefore climate (Davidson & Janssens, 2006; Grace, 2004).

Litter decomposition is governed by climatic conditions both directly, via effects on the composition and activity of decomposer communities, and indirectly via effects on plant litter quality (Swift, Heal, & Anderson, 1979). Most studies focusing on short‐termed direct effects of hydrological change have shown that very low and very high soil moisture slows down decomposition, caused by either a lack of oxygen or by a lack of water that hinders decomposer activity (reviewed in Walter, 2018).

Indirect effects of hydrological change on decomposition, on the other hand, can arise over longer time‐scales, when plant species differ in their response to hydrological conditions due to differing traits among plant species. Different responses of plant species toward changing hydrological conditions based on their traits result in changes in species abundances and consequently functional community composition that can affect decomposition (Figure 2a). Traits might also vary intraspecifically under altered environmental conditions, but this intraspecific trait variation has often been shown to be smaller compared to effects of species turnover on trait variation (Cornwell & Ackerly, 2009; Funk et al., 2017; Hulshof & Swenson, 2010). Numerous studies show that mean traits from trait databases are useful to link, for example, shifts in plant functional community composition under land‐use intensification with ecosystem services or soil functions (e.g., Allan et al., 2015; Boeddinghaus et al., 2019). Showing clear links between shifts in community‐weighted species mean traits and decomposition provides evidence that traits related to hydrology and decomposition vary more between than within species. Predicting the magnitude of effects of environmental change on ecosystem processes mediated by community restructuring will be much easier if these effects can be explained by species‐level mean trait values (cf. McGill, Enquist, Weiher, & Westoby, 2006).

Few studies explicitly tested the consequences of hydrological conditions for community‐weighted mean traits (i.e., response traits sensu Lavorel & Garnier, 2002; only 4% of 568 studies on effects of precipitation for plant functional traits, Griffin‐Nolan et al., 2018). Theoretically, environmental stress should favor resource‐conservative species with lower leaf N and specific leaf area (SLA) and higher leaf dry matter content (LDMC) (Wright et al., 2004). As a result, the community‐weighted mean (CWM: means weighted by the relative abundance or biomass of species) of these traits should change. However, commonly assessed leaf morphological traits such as LDMC and SLA are often only weakly correlated with hydrological conditions (Griffin‐Nolan et al., 2018; Guittar, Goldberg, Klanderud, Telford, & Vandvik, 2016). A positive link between precipitation and CWM SLA has been shown in some studies (Butterfield, Bradford, Munson, & Gremer, 2017; Cornwell & Ackerly, 2009), but not in others (Guittar et al., 2016; Rota et al., 2017).

Functional community restructuring in response to hydrological change can not only lead to shifts in community‐weighted mean traits, but also to changes in functional diversity. Functional diversity takes into account the dissimilarity between species regarding selected traits. It still needs to be elucidated how hydrological conditions shape functional diversity. Functional diversity increases with stress (low soil fertility: Giehl, Jarenkow, & Prinzing, 2015; dryness in grassland: Kimball et al., 2016), with environmental heterogeneity (Bergholz et al., 2017) and with precipitation variability (Gherardi & Sala, 2015). On the other hand, extreme stress should reduce (functional) diversity, because only well‐adapted species with specific trait combinations are able to withstand extreme conditions.

So far, only few studies investigated how CWM traits and functional diversity affect the decomposition of litter from multispecies communities (i. e. effect traits sensu Lavorel & Garnier, 2002; Garnier et al., 2004; Fortunel et al., 2009; Finerty et al., 2016; García‐Palacios, Shaw, Wall, & Hättenschwiler, 2017; Jewell et al., 2017; Santonja et al., 2019). Functional community composition has been suggested to affect decomposition via two different mechanisms: first, according to the biomass‐ratio hypothesis (Grime, 1998), species traits influence ecosystem processes relative to the contribution of a species to the overall biomass of the plant community. Hence, CWM traits should predict ecosystem processes (Garnier et al., 2004). The second hypothesized mechanism underlying effects of functional community shifts on decomposition is niche complementarity: increased functional diversity of litter might enhance decomposition, for example, by offering a broader nutrient spectrum that increases decomposer diversity and activity or because structurally more diverse litter improves microclimatic conditions and habitat availability for decomposers (García‐Palacios et al., 2017; Hättenschwiler, Tiunov, & Scheu, 2005).

CWM of physical and chemical leaf traits (leaf N, LDMC, SLA) is often strongly related to decomposition (García‐Palacios et al., 2017; Jewell et al., 2017). This is in line with single‐species studies that often showed a prominent role of LDMC, but also of SLA and leaf chemicals for decomposition (Freschet, Aerts, & Cornelissen, 2012; Kazakou, Vile, Shipley, Gallet, & Garnier, 2006; Zukswert & Prescott, 2017). It was further shown that functional diversity of litter can modulate decomposition dynamics (Finerty et al., 2016; García‐Palacios et al., 2017; Jewell et al., 2017; Santonja et al., 2018, 2019), but effects of functional diversity were usually weaker than effects of shifts in CWM traits.

Previous studies relating CWM traits or functional diversity with litter decomposition usually used mixed litter with equal proportions of each plant species (with the exception of Garnier et al., 2004 and Fortunel et al., 2009). This does not allow for investigating indirect effects of environmental change on decomposition, caused by changes in species’ abundances. In summary, it remains a key challenge for ecology to predict how changing hydrological conditions affect plant functional community composition via species’ abundance shifts and how resulting shifts in mean functional traits translate into ecosystem processes.

In summary, earlier studies either investigated effects of hydrological change on functional community restructuring or the role of functional community composition for litter decomposition. This study brings both aspects together: We show how functional plant traits can affect the abundance response of plant species to hydrological conditions and therefore how hydrological stress modifies the functional community composition. We further investigate how these community shifts mediate effects of hydrological change on litter decomposition.

In our experimental study, we investigated how changes in hydrological conditions (dryness and wetness) affect the relative abundance (based on biomass proportion or percentage cover) of plant species and consequently CWM traits and functional diversity, with species‐level mean traits obtained from trait databases. We assessed how these shifts in functional vegetation structure affect litter decomposition. We further quantified direct effects of hydrological change on soil respiration, soil decomposer activity, and decomposition of standard litter.

We hypothesized that.

1. Hydrological stress leads to functional community restructuring: Resource‐conservative species (high LDMC, low leaf N and SLA) will be favored, resulting in changes in CWM traits. Functional diversity will decrease under extreme stress (Figure 2a).
2. Shifts in CWM traits and functional diversity via species abundance shifts are related to changes in litter decomposition (Figure 2a).
3. Indirect effects, caused by community restructuring, explain more variation in decomposition than direct effects, that are rooted in decomposer activity.

## 2 MATERIALS AND METHODS

### 2.1 Study site and experimental setup

This mesocosm experiment was conducted in a common garden at the University of Hohenheim, Stuttgart, Southern Germany (48.71°N, 9.19°E; 389 m asl.). Mean annual precipitation from 1981 to 2010 was 718 mm and mean annual temperature was 9.4°C (de Wetter, 2019).

**Figure 1:**
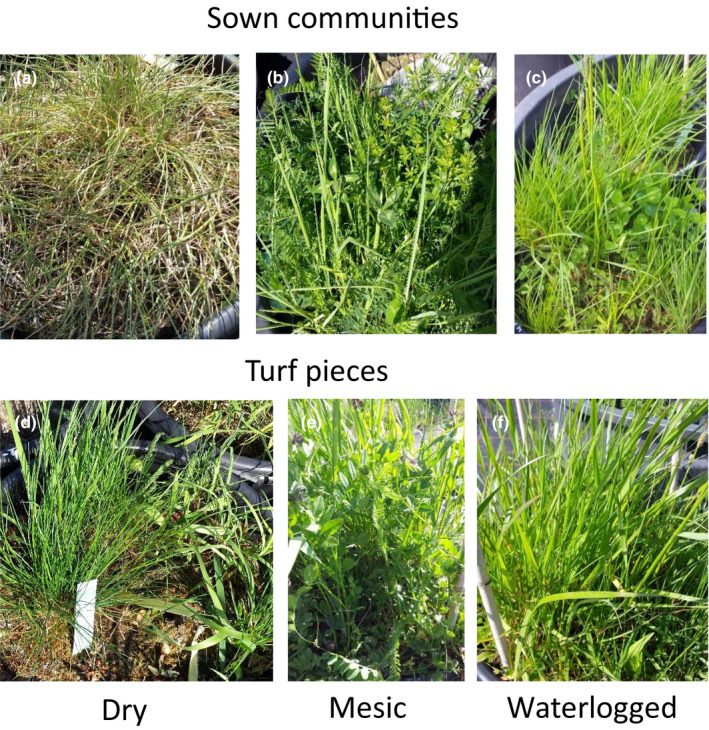
Sown communities (a–c) and turf pieces (d–f) growing under dry (a, d), mesic (b, e), or waterlogged conditions (c, f)

Two different plant communities were used in the study to reveal general mechanisms behind observed responses: sown communities, comprising 15 common grassland species in equal proportions at the start of the experiment (Table 1), and pieces of turf sampled from seminatural hay meadows. While sown communities had an artificial, but precisely defined initial composition, turf communities started from a realistic, but variable initial composition with a fully developed root system. The 120 sown communities (Figure 1a‐c) were established in March 2016 in the greenhouse. We aimed at establishing a density of 90 individuals per pot (6 individuals per species). The number of seeds needed to achieve this density was calculated based on previously measured germination rates of each species and the respective number of seeds was mixed and sown on sandy loam (14% clay, 70% sand, 16% silt; pH 7.88). It was made sure that every species was present in each pot at the beginning of the experiment. Three times during the growing season, nontarget species were removed from sown communities.

**Table 1:** Plant species in sown communities and their associated functional traits, taken from the LEDA and TRY plant trait databases (SLA: specific leaf area [mm^2^ mg^−1^]; LDMC: leaf dry matter content [mg g^−1^]; leaf N: leaf nitrogen content [%])

Species name	SLA	LDMC	leaf *N*
*Achillea millefolium (L.)*	21.3	184.1	1.8
*Alopecurus pratensis (L.)*	26.0	259.3	1.7
*Arrhenatherum elatius (L.)*	30.1	288.5	1.7
*Bellis perennis (L.)*	27.1	113.5	3.2
*Bromus hordeaceus (L.)*	23.4	289.3	2.3
*Centaurea jacea (L.)*	16.1	218.0	3.2
*Crepis biennis (L.)*	31.2	132.5	1.6
*Dactylis glomerata (L.)*	25.8	243.3	2.1
*Festuca rubra agg. (nigrescens)*	21.9	267.0	1.5
*Galium mollugo album (Mill.)*	23.8	168.0	1.6
*Plantago lanceolata (L.)*	21.9	140.2	1.8
*Poa pratensis (L.)*	22.3	308.3	1.7
*Trifolium dubium sibth (L.)*	26.1	199.5	3.2
*Trifolium pratense (L.)*	22.8	218.2	3.9
*Viccia cracca (L.)*	26.2	222.0	3.3

The 105 pieces of turf (0.25 × 0.25 × 0.2 m; Figure 1d‐f) were dug out from four typical, organically managed hay meadows (*Arrhenatherion elatioris*) in Southern Germany on November 19th 2015. Sown communities and turf pieces were established in large pots (20 L, 0.28 m height, 0.35 m diameter) and overwintered in the greenhouse. As turfs originated from different meadows and locations therein, soil type of the upper 0.15–0.2 m differed between turf pieces, while the bottom of the pot was filled with the same soil used for sown communities.

**Figure 2:**
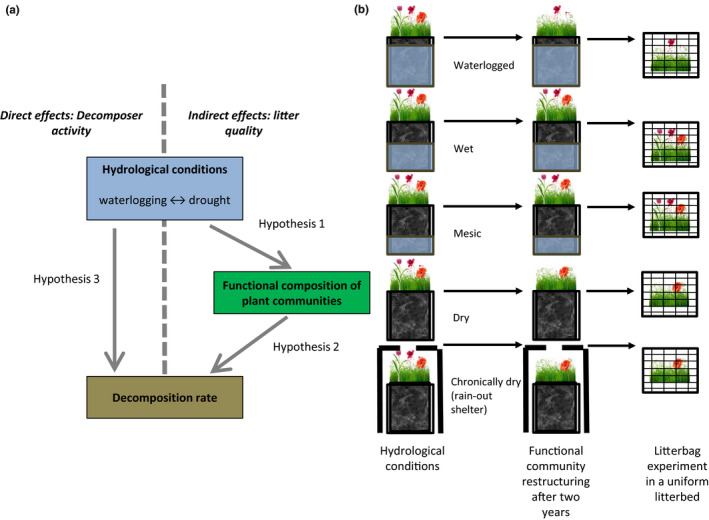
Conceptual figure showing the rationale behind the experiment and the experimental setup for applying water table manipulations. Our study mainly investigates how changes in litter quality caused by shifts in the functional plant community composition after species’ abundance shifts affect decomposition (a, left hand side). To this end, we identify changes in community‐weighted response traits and assess if and how they are linked to decomposition. (b) Hydrological conditions were manipulated by adjusting the water table around the pots using pools with an overflow. Replicate pots of sown communities (*n* = 24 per hydrological condition) and turf pieces (*n* = 21 per hydrological condition) were all placed in separate pools, to prevent pseudoreplication. We hypothesize that hydrological conditions will lead to community restructuring over time that will affect litter decomposition. Dry conditions and chronic drought treatment were pooled for the analyses

All pots were transported to a common garden in April 2016, where we exposed them to different, permanent hydrological conditions by adjusting the water table around them to five levels (Figure 2b). Therefore, pots were placed in overall 120 large pools (275 L) in which the desired water table height was established using overflows. Only one pot of a sown community or turf piece, respectively, was placed into one pool, to avoid pseudoreplication by grouping replicates. Because turf pieces and sown communities were analyzed in different statistical analyses, sometimes a turf piece and a sown community were placed together in one pool. Pools and pots were randomly assigned and arranged.

In the waterlogged treatment (Figure 1a,d), the water table was kept at −5 cm below the soil surface. Average volumetric soil water content (VSW) was 52 ± 1% (mean ± *SE*) in this treatment (see Figure S1). In the wet treatment, the water table height was adjusted to −15 cm (average VSW: 47 ± 1%) and in the mesic treatment to −25 cm (Figure 1b, e; average VSW: 40 ± 1.2%). In the dry treatment, pools contained no water so that pots only received natural rainfall. Under chronic drought conditions, precipitation was additionally reduced by 30% using U‐shaped, inclined, transparent PVC stripes that covered 30% of the area above to intercept approximately 30% of precipitation. The chronic drought treatment, however, did not exert any extra stress on the plants when compared to dry communities (in terms of biomass, community shifts or any other parameter assessed, see also Rai, Klein, & Walter, 2018). We thus pooled both dry levels and refer to them as “dry” hereafter (Figure 1c, f; average VSW in dry treatments 17 ± 0.9%). We included 24 sown communities and 21 turf pieces per hydrological condition.

On 20 June 2016, permanent hydrological manipulations started and were thereafter applied in each year from March until November. The growing season (April 1 until October 31) in 2017 was wetter than the long‐term average from 1981 to 2010 (+86.8 mm of rainfall), while 2018 was exceptionally warm (+2.3 K) and dry (88.8 mm rainfall less during growing season; see Figure S1).

### 2.2 Effects of hydrological condition on plant performance and functional community restructuring (hypothesis 1)

Following ecological concepts that define stress as causing restrictions in biomass production (Grime, 1979), we assessed plant productivity as a proxy for hydrological stress. All plant material was cut 3 cm above the ground in June and October each year, starting in June 2016 for turf pieces and in October 2016 for sown communities. Plant material was dried at 60°C for 4 days and then weighed.

To assess the effects of hydrological stress on community‐weighted mean traits, we determined relative species’ abundances in terms of biomass (for sown communities) or percent cover (for turf pieces). In sown communities, the aboveground plant material harvested in October 2017 was sorted by species to determine species‐specific biomass. For turf pieces, percentage cover of each species for a subset of pots (*N* = 59) was estimated in May 2018, because it was not feasible to reliably sort biomass of many different, often nonflowering species reliably in autumn 2017.

We combined these data on species’ abundances with data on three traits found to play an important role for litter decomposition in previous studies: LDMC, SLA, and leaf N. Species‐level mean values of LDMC and SLA (adult plants, after leaf rehydration, see Cornelissen et al. (2003) were obtained from the LEDA database (Kleyer et al., 2008) and N concentration in dry leaf material from the TRY database (Kattge et al., 2011; see Table 1). We concentrated on living leaf traits because they were shown to be strongly related to “dead” leaf litter traits and are good predictors for litter decomposition (Bakker, Carreño‐Rocabado, & Poorter, 2011; Freschet et al., 2012).

CWM traits for each community were then calculated by weighing species‐level trait values by the abundance of each species. Functional diversity was quantified with the functional dispersion metric FDis (Laliberte & Legendre, 2010). FDis is the mean distance of species to the centroid in multivariate trait space, where both distances and centroid are weighted by the abundance of species. The R package FD was used to calculate CWM and FDis, using lingoes correction (Laliberté, Legendre, & Shipley, 2014).

Whenever the TRY database lacked data on the leaf N content of species in turf communities (data was complete for the 15 species of sown communities), species with <5% cover were ignored for computing CWM N (SLA and LDMC were available for all species). However, when the cover of species without leaf N data exceeded 5%, CWM N and FDis were not computed for this community (16 of the 56 turf communities for which abundance data were assessed). Functional dispersion values based on SLA and LDMC of the 56 turf communities were highly correlated to functional dispersion of the 40 communities also taking into account leaf N (Pearson's correlation coefficient = 0.80) (see Figure S5). We therefore assume no significant impact of leaving out communities with incomplete N data.

To infer on relations between intra‐ and interspecific trait variability in leaf nitrogen (N), we assessed N content of mixed‐ and single‐species plant material after the harvest in October 2017. Mixed community plant material from turf pieces (*N* = 47 (*n* = 9 or 10, 20 in both dry conditions) and sown communities (*N* = 55; with only two replicates for dry communities and otherwise *n* = 10) was analyses as well as material of the two key species *A. pratensis* and *T. pratense* growing in monocultures under the different hydrological conditions (see Table S3, S4 and Methods S2 for results of the side‐experiment investigating plant material from monocultures). Approximately 1 g of a representative sample of dried plant material was milled in a ball mill, and 0.005 g was analyzed for N content with an elemental analyzer (Vario EL, Elementar).

Because not only an increase in average precipitation over midlatitudinal areas, but also an increase in flooding risk is predicted and observed under climate change (IPCC, 2012, 2018), we also investigated how extreme stress pulses relate to milder, longer‐lasting stress of waterlogging. We flooded communities up to 2 cm above the ground for 2 weeks in July 2016 and for 3 weeks in July/August 2017 and 2018 (*n* = 10 for sown communities and *n* = 7 for turf pieces). These pulsed flooding pots were otherwise kept under mesic conditions for the rest of the growing season. Pulsed flooding caused even more extreme community restructuring as compared to permanent waterlogging, but system responses (productivity, decomposition, and functional dispersion) did otherwise not differ between flooded and permanently waterlogged conditions (see Figs. S3, S4 and Table S2 for all results on comparisons between pulsed flooding and waterlogging).

### 2.3 Indirect effect of hydrological conditions on decomposition, mediated by shifts in plant functional community structure

**Figure 3:**
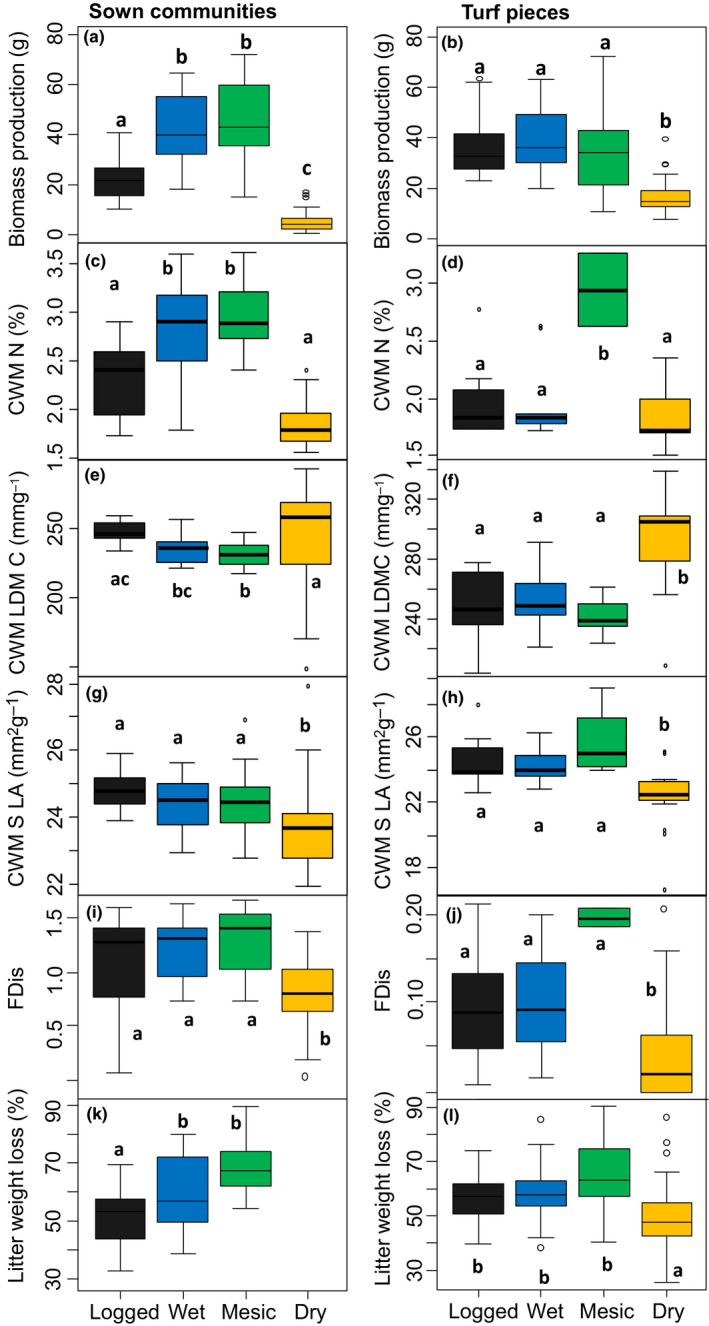
Effects of hydrological conditions on plant performance (a, b), functional community restructuring (c–j) and on the decomposition of litter harvested from communities that had grown under the different hydrological conditions over 2 years (k, l). The left panel shows the results for sown communities with a pre defined species composition, the right panel the results from turf pieces that were dug out from meadows prior to the experiment. Decreased biomass production is seen as an indicator for hydrological stress (sensu Grime, 1979). This hydrological stress caused community restructuring, which was assessed after 2 years (waterlogged = gray; wet = blue; mesic = green; dry = orange). Litter decomposition was assessed on an outside “litterbed” under uniform conditions, to only assess indirect effects of changed litter quality, after communities had grown under different hydrological conditions for almost 2 years. We could not include litterbags for dry sown communities, as biomass production was too low. Different letters show significant differences between hydrological conditions (Tukey's HSD post hoc *p* < .05). Means ± 1 *SE* are shown

To test for indirect effects of litter quality on decomposition, litter in litterbags decomposed under standard common garden conditions. Such a “litterbed” approach is frequently used to investigate solely the indirect effects of initial litter quality on decomposition (Fortunel et al., 2009; Freschet et al., 2012; Quested, Eriksson, Fortunel, & Garnier, 2007 ). We thereby assess the relative decomposability of different litter types and do not attempt to mimic field decomposition. Samples of mixed plant material (5 g) harvested in October 2017 were used to fill litterbags of 0.1 × 0.1 m with a mesh‐size of 1 mm. We did not weigh respective proportions of each plant species, as this was impossible with dried plant material, but we thoroughly mixed litter and took a sample that seemed visually representative for the whole plant community. To avoid using crumbled, broken material, we only filled litterbags with plant material from pots producing more than 7.5 g of dried biomass. As sown communities growing under dry conditions were extremely unproductive (Figure 3a), we could not include litter from dry sown communities. From 13 December 2017 until 17 June 2018, litterbags were placed on a “litterbed” area outside the main experiment and pinned to bare soil with plastic coated wire (*N* = 47 for sown communities (*n* = 14–16) and *N* = 83 for turf pieces (18 from waterlogged 19 from wet, 11 from mesic and 35 from pooled dry communities)). All litterbags were retrieved from the field on 17 June 2018, cleaned, and dried at 60°C for 4 days, and the remaining biomass was weighed to calculate percentage weight loss as a proxy for decomposition.

To test which of the functional community parameters explain decomposition, multiple regressions with all CWM traits and functional dispersion were calculated for sown communities. The significant parameters were then also tested for their explanative power for decomposition of litter from turf communities.

### 2.4 Direct effects of hydrological conditions on decomposition and their relation to indirect effects

To compare indirect litter quality effects on decomposition to direct effects acting via changes in the decomposer activity, we also assessed the decomposition of standard litter, soil respiration, and soil faunal activity under the different hydrological conditions in soil‐filled pots on bare soil. These methods and results are presented in the supporting information (see Methods S1 for methodology and Figs. S2 and S3 for the results). Because indirect effects and direct effects of aboveground litter decomposition were analyzed using different litter and soil types, we only compare the explanatory power (*R* ^2^) of hydrological conditions and do not analyze them together in one model.

### 2.5 Statistical analyses

All data analyses were performed using R 3.5.0 (R Core Team, 2018). Prior to all analyses, homoscedasticity and normality of residuals were checked visually (using package DHARMa for generalized linear models, Hartig, 2018). Data were transformed accordingly whenever necessary.

To test for the fixed effect of hydrological conditions on biomass production, CMW traits, functional dispersion (hypothesis 1), and decomposition (hypothesis 2), we used linear models for sown communities and linear mixed effects models with meadow of origin as a random factor for turf pieces (package lme4; Bates, Maechler, Bolker, & Walker, 2015).

The multiple regressions to relate functional community structure and decomposition (hypothesis 2) were fitted first with all CWM traits and functional dispersion for sown communities and simplified using the step function from R package lmerTest (Kuznetsova, Brockhoff, & Christensen, 2017), which identifies the minimum adequate model using stepwise AIC comparisons. The obtained minimum adequate model structure was then also applied to decomposition of litter from turf communities.

Significance levels were determined using Satterthwaite's method for estimating degrees of freedom for mixed effects models (Kuznetsova et al., 2017) or by comparing models including the factors or interaction term to a model not including these for generalized linear models. Tukey's post hoc tests were calculated in case of significant main effects using the package lsmeans (Lenth, 2016).

## 3 RESULTS

### 3.1 Indirect effects of hydrological stress on decomposition via changes in litter quality (hypothesis 1)

Dryness as well as waterlogging imposed hydrological stress on sown communities (F_3,111_ = 146.1; *p* = .001; *R* ^2^ = .8), shown by significantly reduced biomass production under waterlogged (−52% compared to mesic conditions) and especially under dry conditions (−88% compared to mesic conditions) (Figure 3a) (see Table S1 for all Tukey's HSD post hoc comparisons). Pieces of turf produced less biomass under dry compared to all other conditions (Figure 3b; 53% less than under mesic conditions, while waterlogging did not impose hydrological stress on turf pieces (F_3,104_ = 26.2; *p* < .001; *R* ^2^ = .43).

CWM traits in sown communities significantly responded to hydrological conditions by changes in species abundances. CWM N content (F_3,104_ = 58; *p* < .001; *R* ^2^ = .63) was lower under dry and waterlogged conditions as compared to mesic and wet conditions (Figure 3c). CWM LDMC (F_3,104_ = 5.6; *p* = .001; *R* ^2^ = .14) was higher under dry and waterlogged conditions than under mesic conditions (Figure 3e). CWM SLA (F_3,105_ = 7.5; *p* < .001; *R* ^2^ = .18) was lower under dry when compared to waterlogged and mesic conditions (Figure 3g). CWM N was the most important response trait toward hydrological conditions in sown communities, as hydrological stress increased species abundances of species low in leaf N (*R* ^2^ = .63).

In pieces of turf, CWM of all included traits were significantly affected by hydrological conditions: CWM LDMC (F_3,49_ = 13.6; *p* < .001; *R* ^2^ = .43) was higher and CWM SLA (F_3,49_ = 10; *p* < .001; *R* ^2^ = .36) was lower under dry conditions when compared to all other conditions (Figure 3d, f). Turf pieces had lower CWM *N* (F_3,34_ = 10.3; *p* < .001; *R* ^2^ = .42) content under wet, waterlogged, and dry as compared to mesic conditions (Figure 3h). LDMC and leaf N content were almost equally important response traits toward changing hydrological conditions (*R* ^2^ of .43 and .42, respectively).

Functional dispersion was significantly affected by hydrological conditions in both community types, with lowest FDis under dry conditions (sown communities: F_3,100_ = 11.4; *p* < .001; *R* ^2^ = .25; turf pieces: F_3,35_ = 5.1; *p* = .005; *R* ^2^ = .29; turf pieces only taking into account SLA and LDMC F_3,50_ = 3.39; *p* = .026; see Figure S5). Sown communities revealed lower functional dispersion in dry communities compared to all others (Figure 3), while turf pieces showed significantly lower functional dispersion in dry compared to mesic turf pieces and marginally significant lower functional dispersion in wet compared to dry communities (Figure 3j).

N content of mixed plant material was also significantly affected by hydrological conditions for both, sown communities and turf pieces (F_3,28_ = 4.17; *p* = .009 and F_3,42_ = 6.2; *p* = .001). Both community types had lower N in mixed litter under dry and under waterlogged conditions. Turf pieces had 24% and 52% less N in dry and waterlogged communities, respectively, compared to mesic communities (Figure S6). Sown communities had 45% and 26% less N in dry and waterlogged communities, respectively, compared to mesic communities. Importantly, CWM N calculated using information from trait databases was strongly correlated with measured N content of mixed plant material samples (Pearson's correlation coefficient = 0.74; *p* < .001), hinting toward a large role of community restructuring for N content of plant material (see Figure S6). Contrastingly, N content of monocultures of the two key species, indicating intraspecific trait variation, showed no reaction toward hydrological manipulations and a different response pattern than measured N content of mixed community litter (see Tables S3 and S4).

### 3.2 Indirect effects of litter quality on litter decomposition and its relation to community restructuring (hypothesis 2)

The hydrological conditions under which mixed‐species litter was produced indirectly affected its decomposition (F_2,44_ = 8.87; *p* < .001; *R* ^2^ = .29 for sown communities and F_3,79_ = 5.2; *p* = .002; *R* ^2^ = .16 for turf), with generally slower decomposition when plant communities had experienced hydrological stress, as shown by reductions in biomass production (Figure 3k,l). Litter from sown communities that had grown under waterlogged conditions lost 18.7% (±4.1 *SE*; *p* = .001) less mass than litter grown under mesic conditions. Litter from turf pieces that had grown under dry conditions lost 15.1% (±4.2 *SE*) less mass than litter grown under mesic conditions.

**Figure 4:**
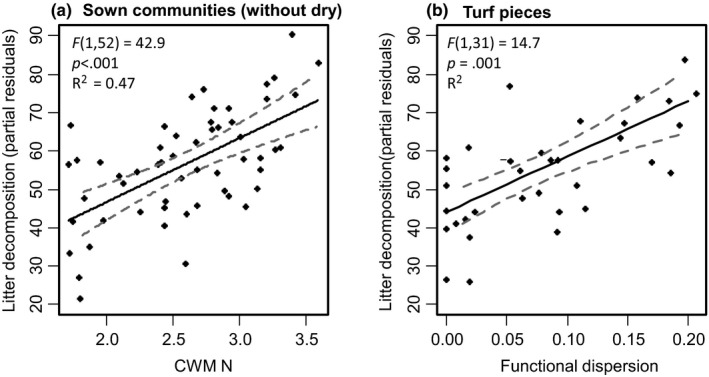
Relation between CWM N in sown communities (a) and functional diversity in turf pieces (b) with decomposition, taking into account the other predictors of most parsimonious regression models. CWM N and functional diversity were the best explaining predictors for decomposition in the respective community type. Partial residuals were calculated including fixed effects of CWM N and FDis, set to their centered mean. Dashed lines show 95% confidence intervals of the models

Multiple regressions were used to identify most important predictors for decomposition of sown community litter. The model with lowest AIC included CWM N and FDis. CWM N (F_1,52_ = 42.9; *p* < .001) and FDis (F_1,52_ = 2.8; *p* = .101) together explained 47% of variance in decomposition, with CWM N alone explaining 44% (Figure 4a). For turf communities, CMW leaf N content and FDis jointly explained 49% of the variance in litter decomposition. FDis alone explained 48% of variance in decomposition for turf pieces (F_1,31_ = 29.2; *p* < .001; Figure 4b).

### 3.3 Comparison of direct and indirect drivers of litter decomposition (hypothesis 3)

Generally, direct effects of hydrological conditions on decomposition processes showed a similar pattern compared with indirect litter quality effects, with reductions in aboveground decomposition and soil respiration under waterlogged and dry conditions (see Figure S2), but no long‐term effects on bait‐lamina‐consumption and thus soil faunal activity during winter. Indirect effects of hydrological conditions on aboveground decomposition mediated by litter quality explained more variance in decomposition than direct effects (multiple *R* ^2^ = .26 for decomposition of sown communities; 0.16 for turf pieces and 0.12 for standard material).

## 4 DISCUSSION

Hydrological stress was imposed on sown plant communities under waterlogged and dry conditions and on turf pieces only by dry conditions, as shown by reduced biomass production. Under hydrological stress, species’ abundance shifts caused the functional composition of plant communities to change toward species with higher CWM LDMC and lower CWM leaf N content (especially in sown communities) and toward lower functional diversity (especially in turf pieces). These stress‐imposed changes in functional community composition rooted in changes in species´ abundances were strongly related to slower litter decomposition. The indirect effects of water conditions on litter decomposition via changes in litter quality are thus expected to amplify decelerating direct effects of hydrological change on C and nutrient cycling. Decomposer activity, as indicated by reduced soil respiration and aboveground decomposition of standard litter, was shown to be reduced under dryness and waterlogging. Tight correlations between calculated CWM N and measured N content of mixed community plant material (see Figure S6) show a large role of community restructuring for chemical and physical trait shifts in litter mixtures. Our study therefore shows for the first time that mean traits from trait databases can have considerable predictive power for ecosystem processes and community responses to hydrological conditions.

### 4.1 Drought and waterlogging impose stress on plant communities and cause functional community restructuring (hypothesis 1)

We found that hydrological stress, as seen by decreases in biomass production, led to community reordering. Not surprisingly, biomass production of young sown communities reacted more sensitively to hydrological stress than established turf pieces. Contrasting to sown communities, established turf communities had a fully developed root system and the opportunity to recruit new species from their seed bank, because they were dug out from natural, long established grassland communities. They could thus respond with complete species turnover toward hydrological change, which might have helped in buffering losses in biomass production.

Hydrological stress resulted in changes in CWM traits, caused by changes in species’ abundances. According to the leaf economics spectrum, stressful environments should favor species with concomitantly low SLA and leaf N and high LDMC (Cornelissen et al., 2003; Wright et al., 2004), because these resource‐conservative species tend to have slower growing, but more stress resistant and more densely packed leaves. This was primarily shown for responses to nutrient stress (Lavorel & Garnier, 2002), but our results partly confirm these assumptions also for hydrological stress: In contrast to the majority of other studies that do not show a clear link between water availability and the most often assessed morphological leaf traits (reviewed in Griffin‐Nolan et al., 2018), we show that CWM N and LDMC can be seen as response traits for hydrological conditions. Our experimental manipulations probably exerted stronger stress and thus caused more pronounced community restructuring than other studies, especially since drought stress avoidance by tap root growth was not possible in our experimental setup. CWM N and CWM LDMC were stronger and more consistent response traits than CWM SLA, which confirms findings showing that LDMC is more closely linked to resource availability (Wilson, Thompson, & Hodgson, 1999) or that CWM SLA is not related to precipitation (Guittar et al., 2016; Rota et al., 2017).

In contrast to other studies, we did not assess species’ traits in situ, except for leaf N content in monocultures of some species. We rather wanted to assess the predictive power of shifts in mean traits based on species turnover. We can therefore only cautiously infer on intraspecific trait variation. We found, however, that CWM N corresponded closely with N content of mixed plant material that we have measured using an elemental analyzer (see Figure S6). Contrastingly, intraspecific shifts in N content of single species showed no reaction toward hydrological manipulations and a different response pattern than measured N content of mixed community litter (see Table S3, S4 and Methods S2 for results of the side‐experiment). This indicates that community species turnover plays a larger role for shifts in litter chemistry of mixed community plant material than intraspecific metabolic shifts. These findings correspond with other studies showing that trait variation between species is usually larger than variation within species (Cornwell & Ackerly, 2009; Funk et al., 2017; Hulshof & Swenson, 2010).

Our results confirm that extreme stress, as seen for dryness, decreases functional diversity. Milder stress, as experienced by sown communities under waterlogged conditions, did not increase functional diversity, which is in contrast to other studies (Gherardi & Sala, 2015; Giehl et al., 2015; Kimball et al., 2016). These studies, however, tested for effects of resource‐related stress, while waterlogging is a nonresource related stress, requiring specific adaptation for survival (Walter, 2018).

### 4.2 Hydrological stress indirectly decelerates litter decomposition, which is linked to functional community restructuring (hypothesis 2)

Litter from stressed communities generally decomposed more slowly than litter from unstressed communities. The observed shifts in CWM traits and functional diversity were tightly linked to litter decomposition. CWM N reacted strongly toward hydrological stress and was a good predictor for litter decomposition, confirming the biomass‐ratio hypothesis. The important role of leaf N is not surprising given that soil microbes are often N limited and that hydrological stress is often also associated with N limitation (Averill & Waring, 2018; Burrit, Tran, Hossain, Kamiya, & Fujiwara, 2017).

Most earlier studies often found CWM traits to play a more prominent role for aboveground litter decomposition than functional diversity (Finerty et al., 2016; García‐Palacios et al., 2017; Jewell et al., 2017). However, our findings for natural, established turf communities confirm the few studies showing a positive relation between functional diversity and litter decomposition (Santonja et al., 2019; Scherer‐Lorenzen, 2008), and thereby also the niche‐complementarity hypothesis. Functionally diverse litter may allow for complementary resource use by offering a broader resource spectrum. This can allow for a higher activity and diversity of decomposers. In our study, increased functional diversity is related to higher N content of mixtures, which hints toward an increased nutrient supply in functionally more diverse litter. Another mechanisms underlying positive effects of functional diversity might be that structurally more diverse litter mixtures may provide more suitable habitats for decomposers or improve litter water holding capacity (Hättenschwiler et al., 2005; Jewell et al., 2017). We cannot completely rule out, however, that the tight relation between functional diversity and litter decomposition might be caused by a sampling effect (increased chance of having a very easily decomposable species in the community when the community is functionally diverse), especially since functional diversity for turf pieces was related to species richness (Pearson's correlation coefficient = 0.61) and also to CWM N.

### 4.3 Indirect effects mediated by functional community restructuring are more important than direct effects for litter decomposition (hypothesis 3)

In accordance to what was suggested by others (Makkonen et al., 2012), we show that indirect effects mediated by litter quality have larger and longer‐lasting effects on decomposition than direct effects: The latter followed our expectations and were in accordance with other studies by revealing depressed soil respiration under waterlogging and decreased aboveground decomposition under both drier and waterlogged conditions (Santonja et al., 2017; Walter, 2018; Figure S2a,b). Indirect effects, however, were larger in magnitude and explained more variation in aboveground decomposition.

## 5 CONCLUSIONS

Our study shows for the first time not only that community‐weighted mean functional traits and functional diversity rapidly change under hydrological stress, caused by shifts in species’ abundances, but also that these shifts in the functional vegetation composition strongly decelerate litter decomposition. We therefore provide evidence for both, the biomass‐ratio and the niche‐complementarity hypotheses, although results regarding the latter need to be interpreted cautiously, because of a tight correlation between functional diversity and CWM leaf N. Both, drier and wetter conditions that are likely to occur more frequently in the future, can therefore severely affect nutrient and C cycling. This is caused by direct effects on decomposer activity, as well as by indirect effects mediated by shifts in litter quality, which are predominantly caused by changes in the relative abundance of already coexisting species.

## CONFLICT OF INTEREST

The authors declare no conflict of interest.

## AUTHOR CONTRIBUTION

**Julia Walter:** Conceptualization (lead); Data curation (lead); Formal analysis (lead); Funding acquisition (lead); Investigation (lead); Methodology (lead); Project administration (lead); Resources (equal); Visualization (equal); Writing‐original draft (lead); Writing‐review & editing (equal). **Carsten M. Buchmann:** Conceptualization (supporting); Data curation (supporting); Formal analysis (supporting); Investigation (supporting); Visualization (equal); Writing‐original draft (supporting); Writing‐review & editing (equal). **Frank M. Schurr:** Conceptualization (supporting); Data curation (supporting); Formal analysis (supporting); Methodology (supporting); Project administration (supporting); Resources (supporting); Visualization (supporting); Writing‐original draft (supporting); Writing‐review & editing (equal). 

## Supporting information

Sup info

## Data Availability

Data are available from the Dryad Digital Repository https://doi.org/10.5061/dryad.jm63xsj71 (Walter, Buchmann, & Schurr, 2020).
