# Supplementary material for: Shifts in plant functional community composition under hydrological stress strongly decelerate litter decomposition
Source: Ecol Evol. 2020 Apr 29;10(12):5712–24. doi: 10.1002/ece3.6310 (PMC7319120; doi:10.1002/ece3.6310)
Supplement: Supplementary file 1 — Sup info [file ECE3-10-5712-s001.docx]

**Supporting information**

**
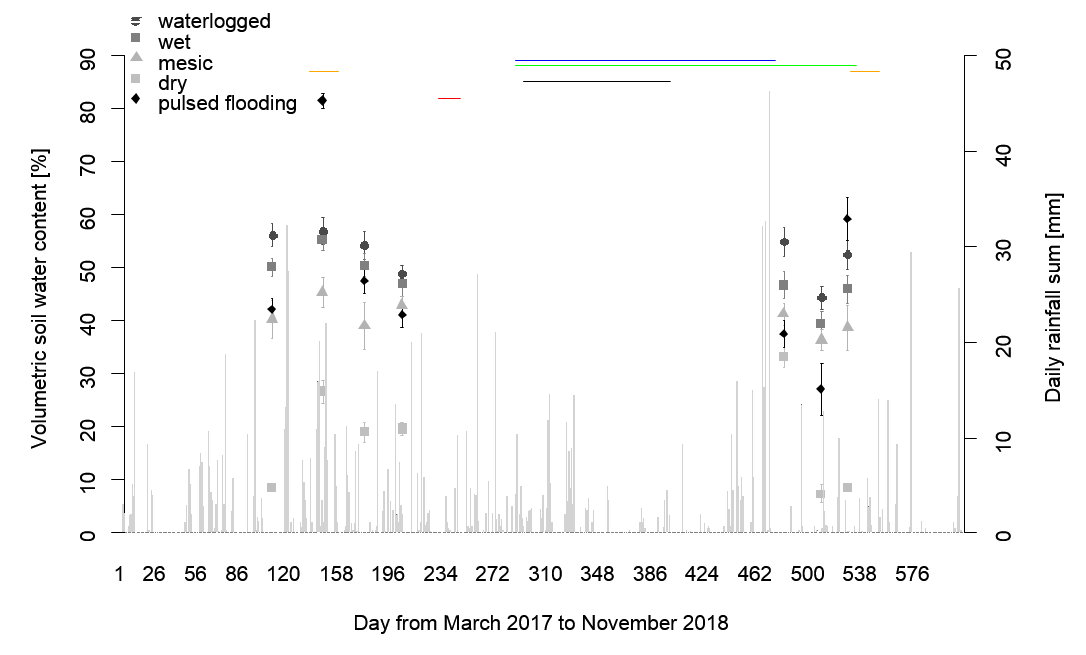
**

**Fig. S1.** Rainfall (grey bars), soil moisture (black diamond: pulsed flooding; dark grey circle: waterlogged treatment; dark grey square: wet treatment; grey triangle: mesic treatment; light grey square: dry treatment) and experimental data acquisition periods (red: harvest of plant material later used for litter bags; blue: duration of experimental litter decomposing in litter bags on an outside area and inside the experiment; black: duration while lamina-bait sticks were placed in soil within the experiment; green: duration when litter bags filled with standard litter where placed within the experiment; orange: pulsed flooding duration and timing in 2017 and 2018). Soil moisture was assessed using a TRD-probe with 20 cm rods in five to seven pots per water availability treatment in experimental communities and meadow pieces.

**Table S1.** Results of Tukey´s HSD post-hoc comparisons that were applied whenever main effects in ANOVA were significant. L=waterlogged treatment, W=wet treatment, M=mesic treatment. FDis= functional dispersion, CMW= community weighted mean; LDMC= leaf dry matter content, SLA=specific leaf area.

|  | **contrast** | **estimate** | **SE** | **df** | **t.ratio** | **p.value** |
| --- | --- | --- | --- | --- | --- | --- |
| Litter weight loss [%] | L - W | -9.1 | 4.3 | 53 | -2.1 | 0.098 |
| of sown community | L - M | -18.3 | 4.3 | 53 | -4.2 | 0.001 |
| litter under standard |  |  |  |  |  |  |
| conditions | W - M | -9.2 | 4.1 | 53 | -2.2 | 0.078 |
|  |  |  |  |  |  |  |
|  |  |  |  |  |  |  |
|  |  |  |  |  |  |  |
| Litter weight loss [%] | L - W | -2.1 | 4.0 | 79 | -0.5 | 0.954 |
| of turf | L - M | -8.4 | 4.6 | 79 | -1.8 | 0.274 |
| litter under standard | L - dry | 6.7 | 3.5 | 79 | 1.9 | 0.238 |
| conditions | W - M | -6.4 | 4.6 | 79 | -1.4 | 0.514 |
|  | W - dry | 8.7 | 3.5 | 79 | 2.5 | 0.063 |
|  | M - dry | 15.1 | 4.2 | 79 | 3.6 | 0.003 |
|  |  |  |  |  |  |  |
| soil respiration (sqrt) | W-L | 0.0 | 0.3 | 0 | 2.6 | 0.043 |
|  | M-L | 0.0 | 0.4 | 0 | 3.9 | <0.001 |
|  | D-L | 0.0 | 0.4 | 0 | 4.6 | <0.001 |
|  | M-W | 0.0 | 0.1 | 0 | 1.2 | 0.618 |
|  | D-W | 0.0 | 0.2 | 0 | 1.6 | 0.392 |
|  | D-M | 0.0 | 0.0 | 0 | 0.2 | 0.997 |
|  |  |  |  |  |  |  |
| weight loss [%] | W-L | -1676.1 | 1303.4 | 56 | -1.3 | 0.576 |
| of standard material [%] (^3) | M-L | -3485.6 | 1331.4 | 56 | -2.6 | 0.054 |
|  | D-L | -426.1 | 1224.1 | 56 | -0.3 | 0.985 |
|  | M-W | -1809.4 | 1120.6 | 56 | -1.6 | 0.379 |
|  | D-W | 1250.0 | 990.7 | 56 | 1.3 | 0.591 |
|  | D-M | 3059.4 | 1027.3 | 56 | 3.0 | 0.022 |
|  |  |  |  |  |  |  |
| biomass | L - W | -1.8 | 0.3 | 111 | -6.2 | <.0001 |
| exp. communities (sqrt) | L - M | -2.0 | 0.3 | 111 | -6.9 | <.0001 |
|  | L - dry | 2.4 | 0.3 | 111 | 9.5 | <.0001 |
|  | W - M | -0.2 | 0.3 | 111 | -0.8 | 0.840 |
|  | W - dry | 4.2 | 0.3 | 111 | 16.8 | <.0001 |
|  | M - dry | 4.5 | 0.3 | 111 | 17.5 | <.0001 |
|  |  |  |  |  |  |  |
| biomass | L - W | 0.0 | 0.1 | 104 | -0.1 | 0.999 |
| turfs (sqrt) | L - M | 0.1 | 0.1 | 104 | 1.3 | 0.590 |
|  | L - dry | 0.8 | 0.1 | 104 | 7.6 | <.0001 |
|  | W - M | 0.2 | 0.1 | 104 | 1.4 | 0.517 |
|  | W - dry | 0.8 | 0.1 | 104 | 7.6 | <.0001 |
|  | M - dry | 0.7 | 0.1 | 104 | 6.7 | <.0001 |
|  |  |  |  |  |  |  |
| N content experimental | W-L | -0.3 | 0.1 | 28 | -2.2 | 0.145 |
| communities | M-L | -0.3 | 0.1 | 28 | -2.5 | 0.075 |
|  | D-L | 0.3 | 0.2 | 28 | 1.5 | 0.471 |
|  | M-W | 0.0 | 0.1 | 28 | -0.3 | 0.987 |
|  | D-W | 0.6 | 0.2 | 28 | 2.7 | 0.049 |
|  | D-M | 0.6 | 0.2 | 28 | 2.9 | 0.032 |
|  |  |  |  |  |  |  |
| N content | W-L | -0.2 | 0.1 | 40 | -2.3 | 0.112 |
| turf | M-L | -0.4 | 0.1 | 41 | -4.1 | 0.001 |
|  | D-L | -0.1 | 0.1 | 40 | -1.4 | 0.524 |
|  | M-W | -0.2 | 0.1 | 41 | -2.1 | 0.158 |
|  | D-W | 0.1 | 0.1 | 41 | 1.3 | 0.573 |
|  | D-M | 0.3 | 0.1 | 41 | 3.4 | 0.007 |
|  |  |  |  |  |  |  |
| species richness | L - W | -0.3 | 0.1 | Inf | -3.1 | 0.012 |
| communities | L - M | -0.3 | 0.1 | Inf | -3.3 | 0.006 |
| (glm) | L - dry | 0.4 | 0.1 | Inf | 4.1 | 0.000 |
|  | W - M | 0.0 | 0.1 | Inf | -0.3 | 0.994 |
|  | W - dry | 0.8 | 0.1 | Inf | 7.6 | <.0001 |
|  | M - dry | 0.8 | 0.1 | Inf | 7.7 | <.0001 |
|  |  |  |  |  |  |  |
| species richness | L - W | 0.1 | 0.1 | Inf | 0.4 | 0.984 |
| turf | L - M | 0.0 | 0.2 | Inf | 0.2 | 0.997 |
| (glm) | L - dry | 1.0 | 0.2 | Inf | 5.6 | <.0001 |
|  | W - M | 0.0 | 0.2 | Inf | -0.1 | 1.000 |
|  | W - dry | 0.9 | 0.2 | Inf | 5.3 | <.0001 |
|  | M - dry | 1.0 | 0.2 | Inf | 4.9 | <.0001 |
|  |  |  |  |  |  |  |
| FDis for exp. | L - W | -0.1 | 0.1 | 100 | -1.4 | 0.481 |
| communities | L - M | -0.2 | 0.1 | 100 | -2.1 | 0.152 |
|  | L - dry | 0.3 | 0.1 | 100 | 2.8 | 0.030 |
|  | W - M | -0.1 | 0.1 | 100 | -0.7 | 0.881 |
|  | W - dry | 0.4 | 0.1 | 100 | 4.5 | <.0001 |
|  | M - dry | 0.5 | 0.1 | 100 | 5.2 | <.0001 |
|  |  |  |  |  |  |  |
| FDis for | L - W | 0.0 | 0.0 | 35 | -0.3 | 0.988 |
| turf | L - M | -0.1 | 0.0 | 35 | -2.2 | 0.151 |
|  | L - dry | 0.0 | 0.0 | 35 | 1.9 | 0.258 |
|  | W - M | -0.1 | 0.0 | 35 | -2.0 | 0.200 |
|  | W - dry | 0.1 | 0.0 | 35 | 2.4 | 0.093 |
|  | M - dry | 0.1 | 0.0 | 35 | 3.4 | 0.010 |
|  |  |  |  |  |  |  |
| CWM LDMC ^2 | L - W | 5920.0 | 2853.9 | 104 | 2.1 | 0.168 |
| exp. communities | L - M | 7486.4 | 2915.3 | 104 | 2.6 | 0.056 |
|  | L - dry | -1408.4 | 2566.5 | 104 | -0.5 | 0.947 |
|  | W - M | 1566.4 | 2853.9 | 104 | 0.5 | 0.947 |
|  | W - dry | -7328.4 | 2496.5 | 104 | -2.9 | 0.021 |
|  | M - dry | -8894.8 | 2566.5 | 104 | -3.5 | 0.004 |
|  |  |  |  |  |  |  |
| CWM LDMC | L - W | -3.5 | 9.2 | 49 | -0.4 | 0.982 |
| turf | L - M | 8.4 | 11.9 | 46 | 0.7 | 0.895 |
|  | L - dry | -43.1 | 8.6 | 50 | -5.0 | <.0001 |
|  | W - M | 11.8 | 11.7 | 50 | 1.0 | 0.743 |
|  | W - dry | -39.7 | 8.3 | 48 | -4.8 | 0.000 |
|  | M - dry | -51.5 | 11.3 | 49 | -4.6 | 0.000 |
|  |  |  |  |  |  |  |
| CWM SLA ^2 exp. | L - W | 23.9 | 14.2 | 104 | 1.7 | 0.339 |
| communities | L - M | 21.9 | 14.5 | 104 | 1.5 | 0.435 |
|  | L - dry | 57.5 | 12.8 | 104 | 4.5 | 0.000 |
|  | W - M | -2.0 | 14.2 | 104 | -0.1 | 0.999 |
|  | W - dry | 33.6 | 12.4 | 104 | 2.7 | 0.039 |
|  | M - dry | 35.6 | 12.8 | 104 | 2.8 | 0.032 |
|  |  |  |  |  |  |  |
| CWM SLA ^2 | L - W | 10.1 | 26.9 | 49 | 0.4 | 0.982 |
| turf | L - M | -64.8 | 34.8 | 48 | -1.9 | 0.258 |
|  | L - dry | 94.2 | 25.3 | 50 | 3.7 | 0.003 |
|  | W - M | -75.0 | 34.4 | 50 | -2.2 | 0.143 |
|  | W - dry | 84.0 | 24.2 | 49 | 3.5 | 0.006 |
|  | M - dry | 159.0 | 33.3 | 50 | 4.8 | 0.000 |
|  |  |  |  |  |  |  |
| CWM N exp. | L - W | -0.5 | 0.1 | 104 | -4.2 | <.0001 |
| communities | L - M | -0.6 | 0.1 | 104 | -5.7 | <.0001 |
|  | L - dry | 0.5 | 0.1 | 104 | 5.1 | <.0001 |
|  | W - M | -0.2 | 0.1 | 104 | -1.6 | 0.402 |
|  | W - dry | 0.9 | 0.1 | 104 | 10.1 | <.0001 |
|  | M - dry | 1.1 | 0.1 | 104 | 11.6 | <.0001 |
|  |  |  |  |  |  |  |
| CWM N | L - W | 0.0 | 0.1 | 33 | 0.1 | 1.000 |
| turf | L - M | -1.0 | 0.2 | 34 | -4.6 | 0.000 |
|  | L - dry | 0.2 | 0.1 | 33 | 1.4 | 0.524 |
|  | W - M | -1.0 | 0.2 | 35 | -4.7 | 0.000 |
|  | W - dry | 0.1 | 0.1 | 33 | 1.4 | 0.519 |
|  | M - dry | 1.2 | 0.2 | 35 | 5.5 | <.0001 |

###### Methods S1. Assessment of direct effects of hydrological conditions on aboveground litter decomposition, soil respiration and lamina-bait consumption

Although much research assessed direct effects of wetness and dryness on decomposition, we included this in our study for being able to relate the significance of the direct to indirect effects. To test for direct effects of hydrological conditions on aboveground litter decomposition, 5 g of standard litter (senesced, intact leaves from a nearby *Platanus × acerifolia*) were filled into 0.05 m x 0.05 m polypropylene litter bags with a mesh size of 0.001 m (N=77). These were placed onto bare soil of all hydrological conditions on December 9^th^ 2017. Half of the bags were retrieved after 157 days, the other half after 249 days. The retrieved bags were cleaned, dried at 60 ° C for four days and the remaining biomass was weighed to calculate percentage weight loss.

To test for direct hydrological effects on heterotrophic soil respiration as a proxy for belowground soil activity during the growing season, we measured soil CO_2_ efflux. It was measured weekly four times shortly after the pulsed flooding ended. We used a LI-8100A coupled to a soil respiration survey chamber of 0.1 m diameter (LI-COR Biosciences, Lincoln, USA) that was placed on permanently installed PVC collars in pots filled with soil, but without vegetation (n=7 per hydrological condition). CO_2_ concentration was measured for 90 seconds, starting 30 seconds after the chamber closed. Soil CO_2_ efflux was calculated based on the exponential equation implemented in LI-8100A.

To test for long-term direct legacy effects of soil moisture on belowground soil faunal activity, we placed 85 bait-lamina sticks (n=17 per hydrological condition) in experimental pots with bare soil from December 20^th^ 2017 until March 25^th^. Bait-lamina sticks are plastic sticks with 16 small holes filled with substrate (the ‘baits’) that can be consumed by the soil fauna. The number of baits consumed is a measure of soil faunal feeding activity (see Kratz, 1998 for details). Since we found in earlier trials that the high water levels in the wet and waterlogged treatment quickly dissolved the baits, we placed the sticks into soil when water manipulations paused over winter.

To test for direct effects of hydrological conditions (fixed effect) on soil respiration (square-root-transformed; hypothesis 1), we used linear mixed effect models, with pot as a random factor to account for temporal pseudo-replication. Effects of hydrological conditions on consumption of lamina baits was tested using generalised linear mixed effects models with a binomial error distribution and pool as a random factor, since some lamina-bait sticks were placed in the same pools. Weight loss of standard material (transformed by the power of three) was tested using a two-factorial linear model with hydrological conditions and duration of placement as factors.

**
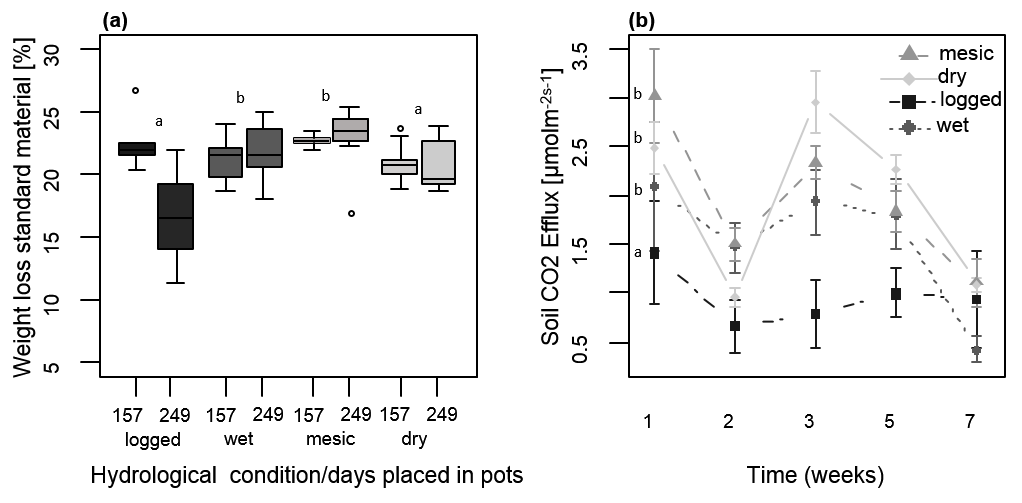
**

**Fig. S2**. Direct effects of hydrological conditions on decomposition of standard litter (a) and soil respiration (b). Both were assessed within the water table manipulation experiment under different hydrological conditions (waterlogged=dark grey; wet=grey; mesic=light grey and dry=off-white). Standard litter was placed on experimental pots for 157 days (from December 2017 until May 2018) and for 249 pots (December until August) and negative effects of hydrological stress got stronger over time. Soil respiration was assessed in August and September 2018. Aboveground decomposition of standard litter was significantly affected by hydrological conditions (F3,56=7.7; p=0.039; R2=0.12), with significantly slower decomposition under waterlogged and dry than under mesic conditions. These effects got more pronounced the longer standard litter decomposed under changed hydrological conditions (Fig. S2 a).

Soil CO2 efflux during summer was also significantly affected by hydrological conditions (F3,34=7.7; p<0.001; R2=0.18) and was on average 49 % lower under waterlogged conditions when compared to mesic conditions (Fig. S2 b). Lamina-bait consumption over winter, when all hydrological treatments paused, did not show any differences between pots subject to different summer hydrological conditions (χ2 (3)=0.64; p=0.43; data not shown).


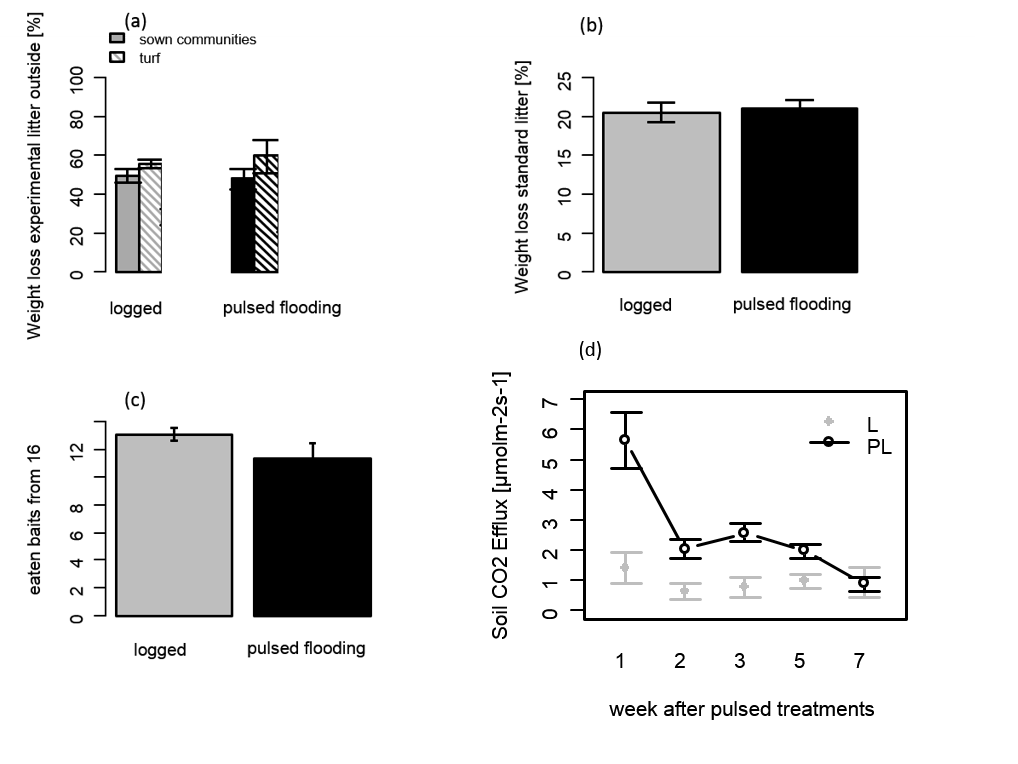


**Fig. S3.** Indirect (a) and direct effects (b-d) of permanently waterlogged and pulsed flooding conditions on litter decomposition and soil processes. a) Litter weight loss of experimental litter from different plant systems (mixed litter of sown communities and turf pieces) that had grown under different water conditions (waterlogged: grey; pulsed flooding: black) during 2017 and was placed outside the experiment under standard conditions during decomposition. b) Litter weight loss of standard litter that was placed under different water conditions inside the experiment over several months. c) Eaten baits from lamina baits sticks places in pots in winter 2017/2018 after pots in which sticks were placed had been under permanently waterlogged (grey, L) or pulsed flooded conditions (black, PL) (to avoid dissolving of the baits under very wet conditions). d) Heterotrophic soil respiration after the pulsed flooding pulse (black, PL) and under permanently waterlogged conditions in August and September 2018. Expectedly, soil respiration was significantly increased after the flooding pulse (F_1,13_= 17.3; p=0.001), but was at the same level as soil respiration of waterlogged soils seven weeks after the pulse (see Supporting Information Fig. S4 and Table S2 for all results on comparisons between pulsed flooding and waterlogging). Soil respiration after pulsed flooding was also considerably higher as soil respiration under mesic conditions (compare Fig. S2), but was at the same level already three weeks after the pulse (when formerly flooded soils were under mesic conditions). Neither soil faunal activity over winter (χ^2^_2_=4.3; p=0.118), nor aboveground decomposition of standard material (Kruskal-Wallis test: χ^2^_1_=0.4; p=0.514) differed between flooded and chronically waterlogged soils.


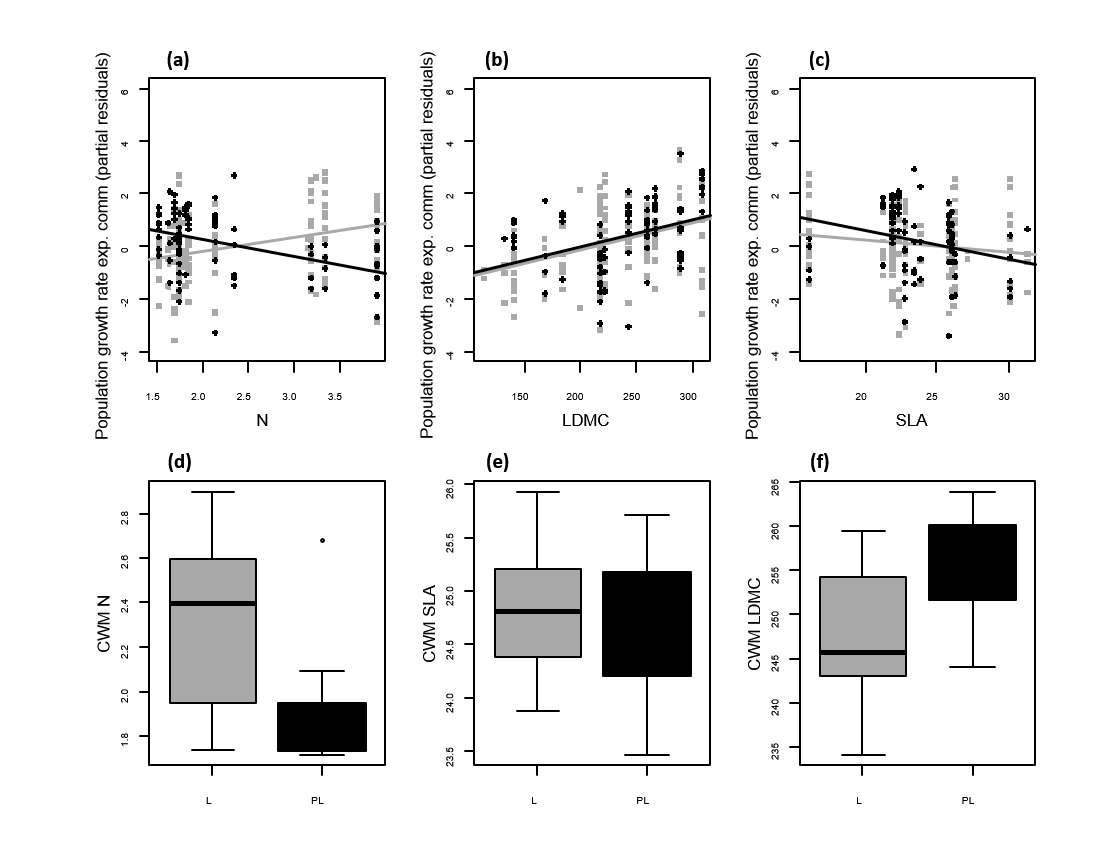

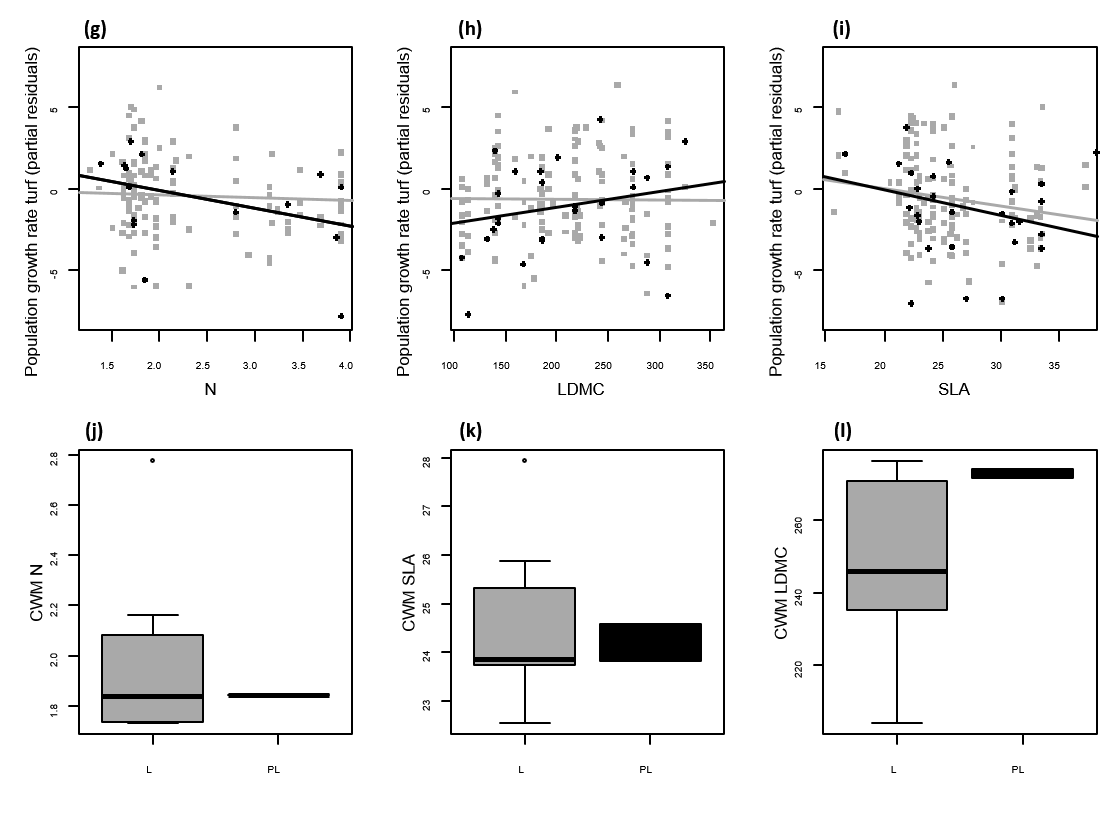


**Fig. S4.** Shifts in species abundances after two growing seasons under different water conditions (grey: waterlogged; black: pulsed flooding) in response to species traits (a-c and g-i) and the associated shifts in community weighted mean functional traits (CWM) (d-f and j-l) for sown communities (a-f) and turf pieces (g-l). Logarithmized abundance of species after two growing seasons is shown for experimental communities (a-c), where all communities started with the same species composition. For turf pieces, the logarithmized response ratio of species´ abundances between 2018 and 2016 is shown (g-i). Partial residuals of population growth rates (exluding the random effects of species identity and pot) are shown in relation to leaf nitrogen content, specific leaf area (SLA) and leaf dry matter content (LDMC) under the different hydrological regimes.

**Table S2.** ANOVA results for pulsed flooding versus permanent waterlogging. Transformation of the response variable is indicated in brackets whenever it was necessary. When generalised linear (mixed effects) models (glm(er)) were performed Chisquare is given instead of an F value (n df= nominator degrees of freedom; r df= residual degrees of freedom; R2=multiple R2 for factor water availability; exp. comm=sown experimental communities).

| **response** | **factor** | **n df** | **r df** | **F/ Chisqu** | **p** | **R2** |
| --- | --- | --- | --- | --- | --- | --- |
| Litter weight loss [%] exp. comm. | **water** | 1 | 26 | 0.1 | 0.749 | 0.00 |
|  |  |  |  |  |  |  |
| Litter weight loss [%] turf | **water** | 1 | 20 | 0.3 | 0.617 | 0.02 |
|  |  |  |  |  |  |  |
| soil respiration (sqrt) | **water** | **1** | **13** | **17.3** | **0.001** | 0.30 |
|  |  |  |  |  |  |  |
| lamina baits consumption |  | 2 |  | 4.3 | 0.118 | 0.03 |
|  |  |  |  |  |  |  |
| weight loss of standard material | water | 1 |  | 0.4 | 0.514 |  |
|  | duration | 1 |  | 3.1 | 0.077 |  |
|  |  |  |  |  |  |  |
| biomass (sqrt) exp. comm | water | 1 | 31 | 2.6 | 0.118 | 0.08 |
|  |  |  |  |  |  |  |
| biomass (sqrt) turf | water | 1 | 25 | 0.1 | 0.768 | 0.00 |
|  |  |  |  |  |  |  |
| N content (log) exp. comm | water | 1 | 18 | 0.1 | 0.778 | 0.00 |
|  |  |  |  |  |  |  |
| N content (log) turf | water | 1 | 11 | 2.3 | 0.160 | 0.12 |
|  |  |  |  |  |  |  |
| species richness exp. comm. | water | 1 |  | 1.6 | 0.201 | 0.04 |
|  |  |  |  |  |  |  |
| species richness turf | water | 1 |  | 0.0 | 0.846 | 0.04 |
|  |  |  |  |  |  |  |
| FDis exp. comm. | water | 1 | 30 | 3.7 | 0.062 | 0.11 |
|  |  |  |  |  |  |  |
| population growth exp. comm | water | 1 | 228 | 0.0 | 0.938 |  |
| log(abundance 2018) | LDMC | 1 | 15 | 1.9 | 0.192 |  |
|  | water:LDMC | 1 | 214 | 0.0 | 0.964 |  |
|  |  |  |  |  |  |  |
|  | water | 1 | 215 | 1.4 | 0.233 |  |
|  | SLA | 1 | 14 | 0.4 | 0.545 |  |
|  | water:SLA | 1 | 204 | 1.3 | 0.263 |  |
|  |  |  |  |  |  |  |
|  | **water** | **1** | **199** | **28.4** | **<0.001** |  |
|  | N | 1 | 13 | 0.0 | 0.929 |  |
|  | **water:N** | **1** | **202** | **34.0** | **<0.001** |  |
|  |  |  |  |  |  |  |
| population growth turf | water | 1 | 152 | 1.5 | 0.228 |  |
| log (abundance 2018/abundance 2016) | LDMC | 1 | 38 | 0.3 | 0.574 |  |
|  | water:LDMC | 1 | 155 | 1.1 | 0.287 |  |
|  |  |  |  |  |  |  |
|  | water | 1 | 167 | 0.1 | 0.791 |  |
|  | SLA | 1 | 46 | 1.9 | 0.174 |  |
|  | water:SLA | 1 | 167 | 0.1 | 0.701 |  |
|  |  |  |  |  |  |  |
|  | water | 1 | 123 | 1.3 | 0.258 |  |
|  | N | 1 | 31 | 0.7 | 0.403 |  |
|  | water:N | 1 | 124 | 1.5 | 0.226 |  |
|  |  |  |  |  |  |  |
|  |  |  |  |  |  |  |
| CWM LDMC exp. communities | **water** | **1** |  | **9.1** | **0.003** | kruskal test |
|  |  |  |  |  |  |  |
| CWM LDMC turf | water | 1 |  | 1.4 | 0.235 | kruskal test |
|  |  |  |  |  |  |  |
| CWM SLA exp. comm. | water | 1 | 30 | 0.6 | 0.430 | 0.02 |
|  |  |  |  |  |  |  |
| CWM SLA turf | water | 1 | 13 | 0.0 | 0.872 | 0.00 |
|  |  |  |  |  |  |  |
| CWM N exp. comm. | **water** | **1** | **30** | **11.1** | **0.002** | 0.27 |
|  |  |  |  |  |  |  |
| CWM N turf | **water** | **1** | **5** | 0.0 | 0.891 | 0.00 |

**Fig. S5.** Functional dispersion (FDis) of turf pieces (a) taking into account leaf N (but discarding communities when species without information on leaf N had substantial cover) and only taking into account SLA and LDMC (b) (which was available for all species). L=waterlogged; W=wet; M=mesic). In both cases, effects of hydrological conditions were significant and dry communities showed significantly lower FDis than mesic communities.

**
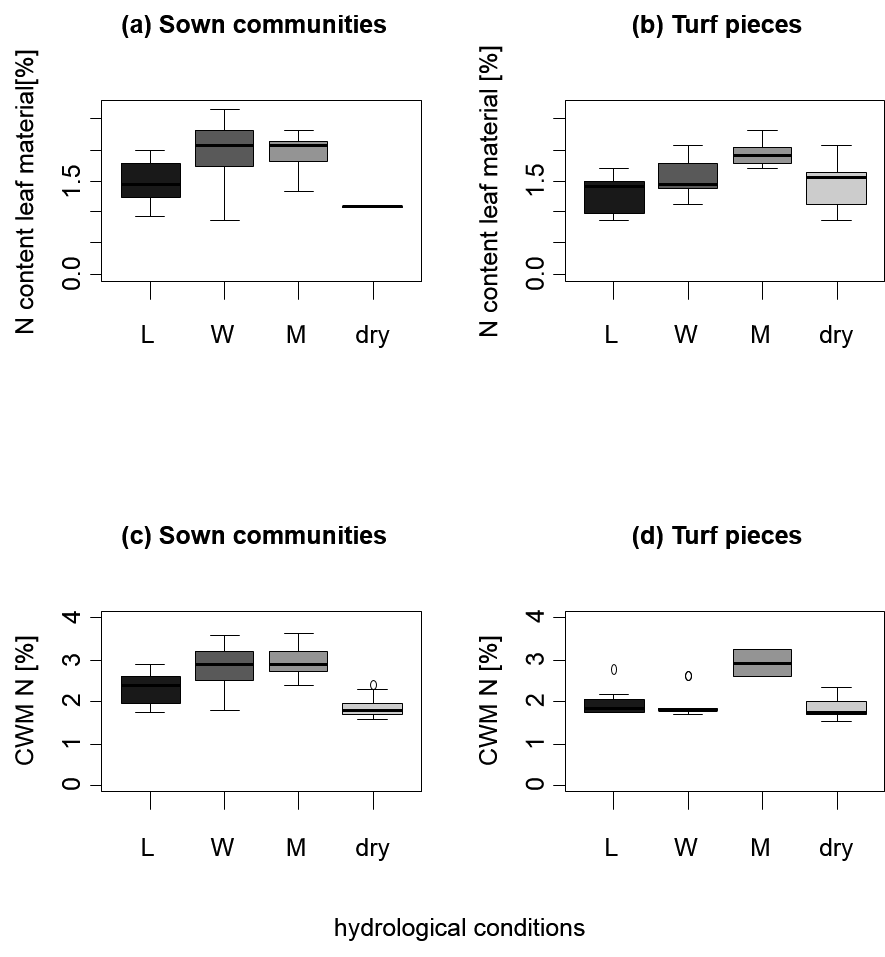
**

**Fig. S6.** Relationship between changes in N content of leaf material measured with an elemental analyser and CWM N content, assessed using species-level mean trait values from trait data bases.

**Methods S2.** In order to investigate how decomposition of single species´ litter relates to decomposition of mixed litter, decomposition of litter from monocultures was assessed for *Alopecurus pratensis* and *Trifolium pratense*. Monocultures were sown in the same pots and soil and at the same density as sown communities. These two species were dominating the sown communities: *A. pratensis* was present in pots of all water conditions (overall 108 out of 120 pots for which data were available) and its average abundance across all conditions was 29 %. *T. pratense* was also present in pots of all water availability treatments (overall 82 of 120 pots), although far less under dry conditions, and its average abundance across all treatments was 21%.

**Table S3**. ANOVA results showing effects of water availability and the different plant systems (experimental communities, turf pieces and monoculture) and their interaction on response variables (left column). Significant interactions (wat:sys) were obtained because monocultures reacted differently from mixed plant communities.

| **response** | **factor** | **n df** | **r df** | **F/ Chisqu** | **p** | **remarks** |
| --- | --- | --- | --- | --- | --- | --- |
| Litter weight loss [%] | **water** | **3** | **154** | **4.2** | **0.007** |  |
| of experimental litter under | **system** | **3** | **154** | **14.2** | **<0.001** |  |
| standard conditions | **wat:sys** | **9** | **154** | **5.7** | **<0.001** |  |
|  |  |  |  |  |  |  |
| biomass (sqrt) | **water** | **3** | **236** | **161.6** | **<0.001** |  |
|  | **system** | **3** | **236** | **37.7** | **<0.001** |  |
|  | **water:sys** | **9** | **236** | **10.3** | **<0.001** |  |
|  |  |  |  |  |  |  |
| N content | **water** | **3** | **91** | **12.5** | **<0.001** |  |
|  | **system** | **3** | **91** | **56.2** | **<0.001** |  |
|  | **water:sys** | **9** | **91** | **2.4** | **0.019** |  |
|  |  |  |  |  |  |  |

**Table S4**. Results of TukeyHSD post-hoc tests for monocultures. Ap= *Alopecurus pratensis*; Tp= *Trifolium pratense*; L= waterlogged; M= mesic; W= wet).

| **Response variable** | **contrast** | **estimate** | **SE** | **df** | **t.ratio** | **p.value** |
| --- | --- | --- | --- | --- | --- | --- |
| Litter weight loss [%] | L,Ap-W,Ap | 3.9 | 7.9 | 22 | 0.5 | 1.000 |
| of monoculture | L,Ap-M,Ap | 8.0 | 9.8 | 22 | 0.8 | 0.990 |
| litter under standard | L,Ap-dry,Ap | 19.0 | 8.6 | 22 | 2.2 | 0.380 |
| conditions | L,Ap-L,Tp | 46.7 | 7.4 | 22 | 6.3 | 0.000 |
|  | L,Ap-W,Tp | 9.2 | 7.9 | 22 | 1.2 | 0.934 |
|  | L,Ap-M,Tp | 28.3 | 7.4 | 22 | 3.8 | 0.018 |
|  | L,Ap-dry,Tp | 47.5 | 9.8 | 22 | 4.8 | 0.002 |
|  | W,Ap-M,Ap | 4.2 | 10.2 | 22 | 0.4 | 1.000 |
|  | W,Ap-dry,Ap | 15.2 | 9.0 | 22 | 1.7 | 0.696 |
|  | W,Ap-L,Tp | 42.8 | 7.9 | 22 | 5.4 | 0.000 |
|  | W,Ap-W,Tp | 5.3 | 8.3 | 22 | 0.6 | 0.998 |
|  | W,Ap-M,Tp | 24.4 | 7.9 | 22 | 3.1 | 0.083 |
|  | W,Ap-dry,Tp | 43.7 | 10.2 | 22 | 4.3 | 0.006 |
|  | M,Ap-dry,Ap | 11.0 | 10.7 | 22 | 1.0 | 0.966 |
|  | M,Ap-L,Tp | 38.6 | 9.8 | 22 | 3.9 | 0.014 |
|  | M,Ap-W,Tp | 1.2 | 10.2 | 22 | 0.1 | 1.000 |
|  | M,Ap-M,Tp | 20.2 | 9.8 | 22 | 2.1 | 0.471 |
|  | M,Ap-dry,Tp | 39.5 | 11.8 | 22 | 3.4 | 0.048 |
|  | dry,Ap-L,Tp | 27.6 | 8.6 | 22 | 3.2 | 0.065 |
|  | dry,Ap-W,Tp | 9.9 | 9.0 | 22 | 1.1 | 0.951 |
|  | dry,Ap-M,Tp | 9.2 | 8.6 | 22 | 1.1 | 0.956 |
|  | dry,Ap-dry,Tp | 28.5 | 10.7 | 22 | 2.7 | 0.190 |
|  | L,Tp-W,Tp | 37.5 | 7.9 | 22 | 4.7 | 0.002 |
|  | L,Tp-M,Tp | 18.4 | 7.4 | 22 | 2.5 | 0.258 |
|  | L,Tp-dry,Tp | 0.9 | 9.8 | 22 | 0.1 | 1.000 |
|  | W,Tp-M,Tp | 19.1 | 7.9 | 22 | 2.4 | 0.281 |
|  | W,Tp-dry,Tp | 38.4 | 10.2 | 22 | 3.8 | 0.020 |
|  | M,Tp-dry,Tp | 19.3 | 9.8 | 22 | 2.0 | 0.530 |
|  |  |  |  |  |  |  |
| biomass | L,Ap-W,Ap | -0.1 | 0.9 | 21 | -0.1 | 1.000 |
| monocultures (log) | L,Ap-M,Ap | 0.4 | 0.9 | 21 | 0.5 | 1.000 |
|  | L,Ap-dry,Ap | 1.7 | 0.8 | 21 | 2.2 | 0.365 |
|  | L,Ap-L,Tp | 0.0 | 0.9 | 21 | 0.0 | 1.000 |
|  | L,Ap-W,Tp | -1.1 | 1.0 | 21 | -1.1 | 0.952 |
|  | L,Ap-M,Tp | -3.2 | 0.9 | 21 | -3.6 | 0.027 |
|  | L,Ap-dry,Tp | 2.7 | 0.8 | 21 | 3.6 | 0.029 |
|  | W,Ap-M,Ap | 0.5 | 0.9 | 21 | 0.6 | 0.999 |
|  | W,Ap-dry,Ap | 1.8 | 0.8 | 21 | 2.4 | 0.293 |
|  | W,Ap-L,Tp | 0.1 | 0.9 | 21 | 0.2 | 1.000 |
|  | W,Ap-W,Tp | -0.9 | 1.0 | 21 | -1.0 | 0.974 |
|  | W,Ap-M,Tp | -3.1 | 0.9 | 21 | -3.5 | 0.036 |
|  | W,Ap-dry,Tp | 2.8 | 0.8 | 21 | 3.8 | 0.021 |
|  | M,Ap-dry,Ap | 1.3 | 0.8 | 21 | 1.7 | 0.686 |
|  | M,Ap-L,Tp | -0.4 | 0.9 | 21 | -0.4 | 1.000 |
|  | M,Ap-W,Tp | -1.5 | 1.0 | 21 | -1.5 | 0.793 |
|  | M,Ap-M,Tp | -3.6 | 0.9 | 21 | -4.1 | 0.010 |
|  | M,Ap-dry,Tp | 2.3 | 0.8 | 21 | 3.1 | 0.088 |
|  | dry,Ap-L,Tp | -1.7 | 0.8 | 21 | -2.2 | 0.389 |
|  | dry,Ap-W,Tp | -2.8 | 0.9 | 21 | -3.2 | 0.074 |
|  | dry,Ap-M,Tp | -4.9 | 0.8 | 21 | -6.5 | 0.000 |
|  | dry,Ap-dry,Tp | 1.0 | 0.6 | 21 | 1.7 | 0.700 |
|  | L,Tp-W,Tp | -1.1 | 1.0 | 21 | -1.1 | 0.944 |
|  | L,Tp-M,Tp | -3.2 | 0.9 | 21 | -3.7 | 0.025 |
|  | L,Tp-dry,Tp | 2.7 | 0.8 | 21 | 3.6 | 0.032 |
|  | W,Tp-M,Tp | -2.1 | 1.0 | 21 | -2.2 | 0.409 |
|  | W,Tp-dry,Tp | 3.8 | 0.9 | 21 | 4.4 | 0.006 |
|  | M,Tp-dry,Tp | 5.9 | 0.8 | 21 | 7.8 | <.0001 |
|  |  |  |  |  |  |  |
| N content single | L,Ap-W,Ap | 0.4 | 0.3 | 20 | 1.1 | 0.956 |
| plants | L,Ap-M,Ap | 0.5 | 0.3 | 20 | 1.5 | 0.780 |
|  | L,Ap-dry,Ap | 0.3 | 0.3 | 20 | 1.2 | 0.921 |
|  | L,Ap-L,Tp | 1.5 | 0.3 | 20 | 5.0 | 0.002 |
|  | L,Ap-W,Tp | 1.5 | 0.3 | 20 | 5.2 | 0.001 |
|  | L,Ap-M,Tp | 2.4 | 0.3 | 20 | 8.1 | <.0001 |
|  | L,Ap-dry,Tp | 2.2 | 0.3 | 20 | 8.5 | <.0001 |
|  | W,Ap-M,Ap | 0.1 | 0.3 | 20 | 0.3 | 1.000 |
|  | W,Ap-dry,Ap | 0.0 | 0.3 | 20 | 0.2 | 1.000 |
|  | W,Ap-L,Tp | 1.1 | 0.3 | 20 | 3.4 | 0.050 |
|  | W,Ap-W,Tp | 1.2 | 0.3 | 20 | 3.6 | 0.032 |
|  | W,Ap-M,Tp | 2.1 | 0.3 | 20 | 6.2 | 0.000 |
|  | W,Ap-dry,Tp | 1.9 | 0.3 | 20 | 6.2 | 0.000 |
|  | M,Ap-dry,Ap | 0.1 | 0.3 | 20 | 0.6 | 0.999 |
|  | M,Ap-L,Tp | 1.0 | 0.3 | 20 | 3.4 | 0.044 |
|  | M,Ap-W,Tp | 1.1 | 0.3 | 20 | 3.7 | 0.027 |
|  | M,Ap-M,Tp | 2.0 | 0.3 | 20 | 6.6 | <.0001 |
|  | M,Ap-dry,Tp | 1.8 | 0.3 | 20 | 6.8 | <.0001 |
|  | dry,Ap-L,Tp | 1.2 | 0.3 | 20 | 4.5 | 0.004 |
|  | dry,Ap-W,Tp | 1.2 | 0.3 | 20 | 4.8 | 0.002 |
|  | dry,Ap-M,Tp | 2.1 | 0.3 | 20 | 8.2 | <.0001 |
|  | dry,Ap-dry,Tp | 1.9 | 0.2 | 20 | 8.8 | <.0001 |
|  | L,Tp-W,Tp | 0.1 | 0.3 | 20 | 0.2 | 1.000 |
|  | L,Tp-M,Tp | 0.9 | 0.3 | 20 | 3.2 | 0.075 |
|  | L,Tp-dry,Tp | 0.8 | 0.3 | 20 | 2.9 | 0.121 |
|  | W,Tp-M,Tp | 0.9 | 0.3 | 20 | 2.9 | 0.121 |
|  | W,Tp-dry, Tp | 0.7 | 0.3 | 20 | 2.6 | 0.198 |
|  | M,Tp-dry,Tp | 0.2 | 0.3 | 20 | 0.6 | 0.998 |
|  |  |  |  |  |  |  |
